# Integrated oral-gut microbiota therapy: a novel perspective on preventing bacterial translocation for systemic disease management

**DOI:** 10.3389/fcimb.2025.1641816

**Published:** 2025-07-28

**Authors:** Jie Zhu, Ziyi Jiang, Fangli Yu, Linglin Gao, Xiaomei Wang, Qiang Wang

**Affiliations:** ^1^ Institute of Infection, Immunology and Tumor Microenvironment, Wuchang Hospital Affiliated to Wuhan University of Science and Technology, Medical College, Wuhan University of Science and Technology, Wuhan, China; ^2^ Department of Gynecology, Maternal and Child Health Hospital of Hubei Province Graduate Joint Training Base, School of Medicine, Wuhan University of Science and Technology, Wuhan, China

**Keywords:** oral microbiota, gut microbiota, oral-gut axis, systemic diseases, bacterial translocation, integrative therapy

## Abstract

Oral dysbiosis increases the risk of oral diseases and systemic diseases, with many related conditions overlapping with systemic diseases triggered by gut dysbiosis. Studies have shown that the oral cavity serves as an endogenous reservoir for gut microbial strains, influencing the homeostasis of both oral and gut microbiota through interactions involving bacterial translocation, microbial metabolites, immune cells, and inflammatory factors. In specific disease contexts, certain microbial communities [e.g., *Porphyromonas gingivalis (P.g)*, *Fusobacterium nucleatum (F.n)*], metabolites (e.g., short-chain fatty acids, gingipains), ligands (e.g., lipopolysaccharides, peptidoglycans), or host responses may vary. However, substantial evidence has firmly established the central role of microbiota in oral-gut crosstalk. These findings position the oral-gut axis as a potential causal mechanism linking systemic diseases. Compared with healthy non-cancer subjects, cancer patients exhibit significant differences in oral microbial abundance and diversity. For instance, *F.n* is associated with an increased risk of colorectal cancer(CRC), while *Oribacterium* and *Fusobacterium* may serve as potential biomarkers for hepatocellular carcinoma. Notably, oral pathogens or their metabolites can translocate along the oral-gut axis or due to certain oral activities (e.g., toothbrushing, tooth extraction), contributing to the initiation and progression of inflammation and tumorigenesis. For example, *P.g* can accumulate in the liver, where its fimbrial protein FimA binds to Toll-like receptor 2 (TLR2), complement receptor 3 (CR3), and CXC-chemokine receptor 4 (CXCR4), triggering various immune responses that promote the development of non-alcoholic fatty liver disease(NAFLD). This review systematically summarizes recent advances in understanding the role of the oral microbiota and the oral-gut axis in systemic diseases, along with their underlying pathological mechanisms. It particularly highlights the translational value of integrating oral and gut microbiota research, offering novel insights for the prevention and precision treatment of systemic disorders. The unique and heterogeneous microbiota within the oral microbiota and the oral-gut axis may serve as novel diagnostic biomarkers or therapeutic targets for diseases associated with oral and gut dysbiosis.

## 1 Introduction

The oral microbiota, comprising over 700 microbial species, represents the second most abundant and diverse microbial community in the human body (Suárez et al., 2020). The oral cavity’s complex anatomical structures—including the gingival sulcus, tongue, saliva, and teeth—create heterogeneous microenvironments with varying oxygen levels and pH, leading to distinct niche-specific microbial compositions. Oral microbiota plays a crucial role in maintaining oral homeostasis by inhibiting the colonization of pathogenic microbiota and stimulating the synthesis of saliva antimicrobial components (such as IgA and defensins) in the oral microbiota. However, risk factors like smoking, alcohol consumption, or poor oral hygiene can disrupt this equilibrium, promoting the overgrowth of opportunistic pathogens (e.g., *P.g*, *F.n*). These pathogens enhance virulence factor production (e.g., LPS, peptidoglycan, gingipains), which degrade intercellular adhesion molecules, compromise epithelial integrity, and facilitate bacterial invasion into deeper tissues and systemic circulation (Lamont et al., 2018; Hajishengallis and Chavakis, 2021). Consequently, this process may trigger systemic inflammation, metabolic dysregulation, and bacteremia, enabling bacterial dissemination to distant organs and contributing to extra-oral diseases.

Oral pathogens and their metabolites (e.g., LPS, bacterial toxins) can translocate to the gut via hematogenous or enteral routes, compromising gut barrier function and disrupting microbial balance. Such dysbiosis may exacerbate intestinal permeability (“leaky gut”), endotoxemia, and systemic dissemination of microbial byproducts, thereby inducing systemic pro-inflammatory state (Fleetwood et al., 2017). Meanwhile, these factors may also disrupt immune homeostasis by modulating gut immunity, including increased helper T cell 1 (Th1) and Th17 responses alongside reduced regulatory T cell (Treg) activity, thereby promoting systemic inflammatory responses (Pacheco-Yanes et al., 2023). For instance, PD-associated oral dysbiosis alters microbial metabolite profiles (e.g., reduced SCFAs, elevated bile acids and aromatic amino acids), which may further impair gut barrier integrity and promote metabolic and neuroinflammatory disorders (Yamazaki, 2023). Interestingly, gut dysbiosis can indirectly influence oral health, suggesting bidirectional crosstalk along the oral-gut axis. Thus, the oral-gut axis serves as a critical pathway linking oral dysbiosis to systemic pathologies. Notably, key pathogens such as *P.g* and *F.n* have been demonstrated to contribute to the development of various systemic diseases via this axis. Strikingly, the translocation and colonization of other oral commensals—such as *Aggregatibacter actinomycetemcomitans*(*A.a*) and *Treponema denticola (T.denticola)*—exacerbate intestinal permeability and promote chronic liver disease (Åberg and Helenius-Hietala, 2022; Xi et al., 2024), while *Prevotella intermedia* and *A.a* show significant associations with autoimmune disorders like arthritis (Konig et al., 2016; Lorenzo et al., 2019). These findings suggest that a systemic imbalance in the oral-gut microbial network, rather than individual pathogens, may be the central mechanism driving systemic diseases. In this review, we synthesize current evidence on the oral-gut axis as a unifying framework for understanding the role of microbiota in systemic disorders. We emphasize the need for integrated therapeutic strategies that simultaneously target both oral and gut ecosystems to mitigate local and systemic inflammation.

## 2 Factors affecting the homeostasis of oral microbiota

**Figure 1:**
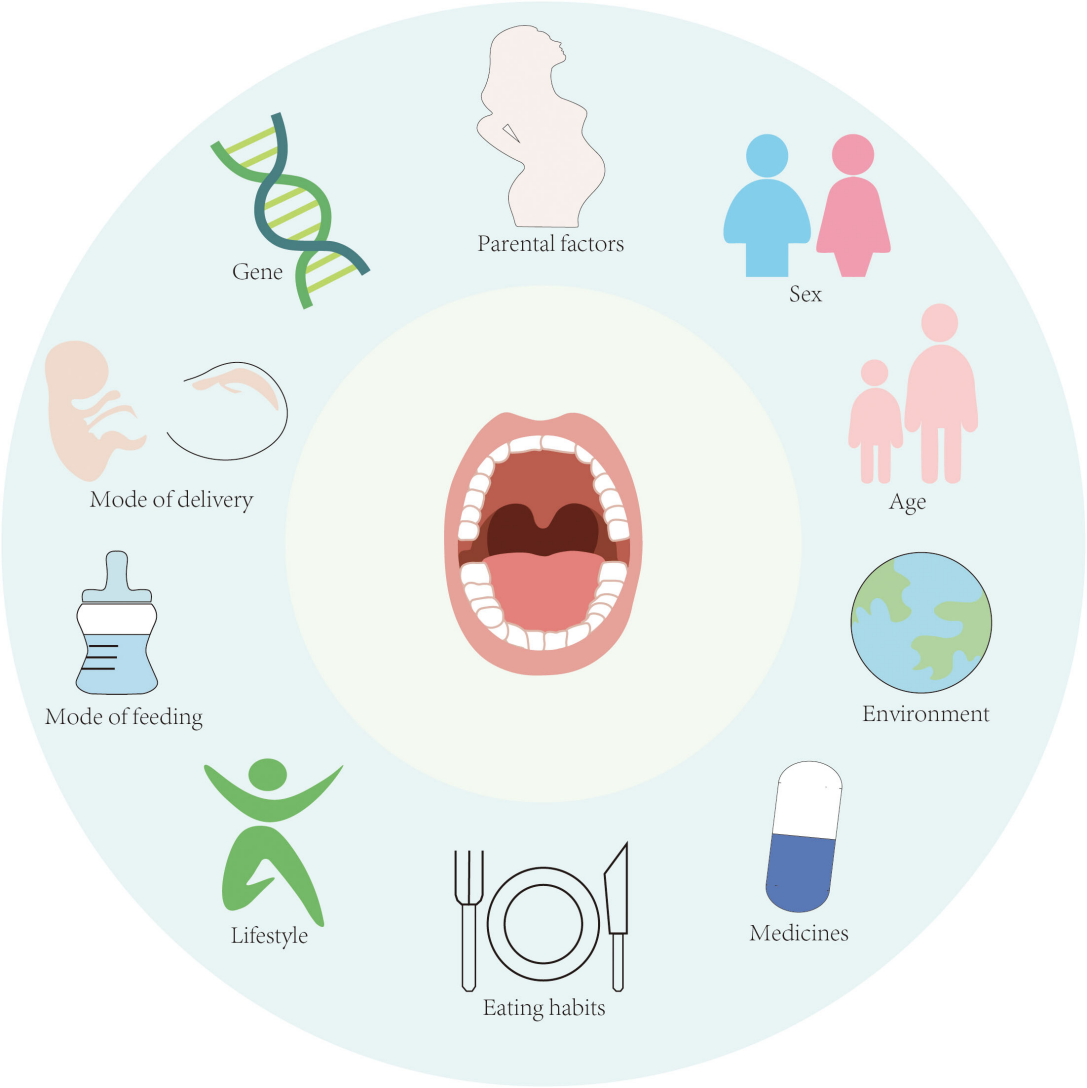
Factors influencing the homeostasis of oral microbiota.

The composition of the oral microbiota is shaped by a complex interplay of intrinsic and extrinsic factors, including genetic predisposition, maternal influences, birth mode, gender, age, environmental exposures, lifestyle habits, and dietary patterns (**Figure 1**). These factors collectively contribute to the interindividual variation observed in oral microbiota (Stankevic et al., 2024). Emerging evidence suggests that certain health-associated oral microbiota may exhibit heritability. In a seminal study by Corby et al., investigators examined 48 monozygotic (MZ) and 54 dizygotic (DZ) twin pairs to assess genetic contributions to oral microbiota colonization. Their analysis estimated heritability at 52%, with non-shared environmental factors accounting for the remaining 48% of variation (Corby et al., 2005). However, conflicting findings have been reported in studies comparing salivary microbiota between MZ and DZ twins, where no significant differences were detected (Ooi et al., 2014; Du et al., 2017). These discrepancies suggest that environmental influences and epigenetic modifications may outweigh genetic determinants in shaping the oral microbiota. Notably, external factors such as lifestyle practices, social structures, and shared environments appear to exert stronger effects than host genetics (Willis et al., 2022). This perspective is supported by Willis et al.’s research demonstrating greater microbial similarity among family members compared to unrelated individuals.

Environmental factors further modulate oral microbiota composition through their impact on host behaviors and dietary patterns. Cross-population studies reveal distinct microbial signatures: *Neisseria* spp. predominates in healthy Chinese populations, *Veillonella* is prevalent among Canadians, while *Prevotella* dominates in Qatari populations (Murugesan et al., 2020; Nearing et al., 2020; Cheung et al., 2022). Cheung et al.’s 16S rRNA sequencing analysis of healthy Chinese adults identified significant associations between oral microbial alpha diversity and both age and gender (Cheung et al., 2022). These age-related microbial shifts may reflect physiological changes including tooth eruption patterns and age-dependent alterations in hormonal and immune system function. Notably, elevated cortisol levels in gingival crevicular fluid have been observed in periodontal disease patients (Rai et al., 2011), and *in vitro* studies demonstrate that cortisol exposure enhances metabolic activity of *F.n* and *Leptotrichia goodfellowii* in dental plaque (Duran-Pinedo et al., 2018). Aiko et al. further established significant correlations between serum cortisol/dehydroepiandrosterone sulfate levels and periodontal disease severity (Ishisaka et al., 2008). Physical and chemical perturbations from dietary intake, oral hygiene practices, and mucosal turnover continuously modify biofilm composition. The dynamic nature of oral biofilms is exemplified by the ecological succession from facultative anaerobes in early colonization to strictly anaerobic species as biofilm matures and oxygen availability decreases (Velsko et al., 2019). Consequently, distinct microbiota characterize developing versus mature biofilms, with biofilm-environment interactions playing a pivotal role in maintaining oral microbial homeostasis.

The stability of the oral ecosystem is maintained through tripartite interactions among microbiota, mucosal immune cells, and epithelial barriers. A balanced oral microbiota contributes to host health through reciprocal relationships between microbial species and between microbiota and their host (Kamada et al., 2013; Şenel, 2021). Commensal bacteria stimulate host immune responses that produce antimicrobial peptides while directly inhibiting pathogen colonization. Furthermore, beneficial symbionts modulate epithelial cell morphology and intercellular junction gene expression to preserve mucosal integrity (Takahashi et al., 2019; La Rosa et al., 2020). Antimicrobial peptides exemplify this dual function by both combating pathogens and enhancing tight junction gene expression. The oral immune network relies on sustained cytokine and chemokine signaling. Oral epithelial cells express an array of pattern recognition receptors (TLRs, NOD1, NOD2, PARs) that coordinate microbial responses and inflammatory mediator production. For instance, NOD1-mediated recognition of Gram-negative bacterial components triggers downstream signaling cascades that induce antimicrobial peptides (hBD-2) and pro-inflammatory cytokines (IL-6, IL-8, TNF-α) (Groeger and Meyle, 2019). Salivary components including IgA, mucins, and antimicrobial peptides form a critical chemical barrier, with secretory IgA particularly important for inhibiting microbiota adhesion to oral surfaces (Baker et al., 2017). Collectively, these interdependent systems maintain oral microbiota equilibrium.

## 3 The oral-gut axis: Interaction between oral and gut microbiota

### 3.1 Oral microbiota dysbiosis affects gut homeostasis and causes systemic diseases

Traditionally, the gastrointestinal tract maintains physiological segregation between oral and gut microbiota through multiple defense mechanisms collectively termed the “oral-gut barrier”. This barrier system comprises chemical defenses (gastric acid and bile acids), host pattern recognition receptors, and colonization resistance conferred by indigenous gut microbiota. Within this paradigm, oral-to-gut microbiota translocation was historically viewed as a pathological aberration. However, emerging evidence challenges this notion, demonstrating that substantial quantities of oral microbiota—both as free-living organisms and within keratinocyte complexes—routinely enter the digestive tract through dietary intake. Salivary components (including water, lipids, and mucins) provide crucial protection, enabling these microorganisms to survive the harsh gastric environment and persist in the gastrointestinal tract. Abdelary et al.’s sodA sequence analysis definitively established the oral cavity as an endogenous source of gut microbiota, confirming that oral-gut microbiota translocation represents a widespread physiological phenomenon in healthy individuals (Schmidt et al., 2019; Abdelbary et al., 2022).

This translocation process becomes particularly pronounced when gastric acid and bile acid barriers are compromised or when microbiota develop resistance to these defenses, as demonstrated by Jackson and Boll’s research (Boll et al., 2012; Jackson et al., 2016). Clinical observations reveal significantly elevated levels of oral-derived bacteria (including *Streptococcus*, *Veillonella*, and *Haemophilus* spp.) in the intestines of long-term proton pump inhibitor users and inflammatory bowel disease (IBD) patients compared to healthy controls (Kitamoto et al., 2020b; Imai et al., 2021; Kitamoto and Kamada, 2022). Notably, the gut microbiota of IBD patients exhibits marked “oralization,” acquiring compositional features resembling oral microbiota. Certain oral pathogens (e.g., *P.g*) can survive the acidic gastric environment and traverse the gastric barrier (Walker et al., 2018). The oral microbiota may also disseminate to the gut via hematogenous or lymphatic routes. For instance, during dental procedures, increased oral epithelial permeability, or food ingestion, oral microbes may infiltrate and spread to extraoral sites. *F.n*, a Gram-negative oral anaerobe, exhibits strong binding affinity to vascular endothelium and modulates barrier permeability, potentially facilitating hematogenous dissemination—even transplacentally—and promoting co-invasion of commensal bacteria (e.g., *Escherichia coli*) into circulation. Finally, some oral pathogens carrying virulence factors that inhibit phagolysosome formation can invade dendritic cells and macrophages, hijacking these immune cells as “Trojan horses” for intestinal migration (Hajishengallis, 2015).

Bacterial translocation or their metabolites can disrupt the homeostasis of the gut microbiota and compromise intestinal barrier function, leading to dysbiosis (Kitamoto et al., 2020c; Mo et al., 2022). Oral microbiota colonize the gut by triggering intestinal inflammation, impairing colonization resistance of the gut microbiota, and enhancing resistance to the gastrointestinal chemical barrier, thereby disrupting host-microbiome homeostasis (Kim et al., 2017; Ducarmon et al., 2019). Intestinal inflammation is closely linked to gut dysbiosis, as inflammation provides an ecological niche for ingested oral bacteria while impairing pattern recognition receptors (PRRs) of gut immunity, resulting in reduced antimicrobial peptide production and diminished pathogen clearance (Larabi et al., 2020). Studies have found that periodontal pathogens (e.g.,*P.g*) produce an interspecies quorum-sensing signal called autoinducer-2 (AI-2), which modulates gut microbiota composition by promoting the growth of Firmicutes and increasing the *Firmicutes/Bacteroidetes* (*F/B*) ratio (Thompson et al., 2015; du Teil Espina et al., 2019). Certain oral microbes exhibit distinct metabolic pathways compared to gut commensals, enhancing their adaptability and competitive advantage in the inflammatory intestinal environment (Kitamoto et al., 2020a). Periodontal pathogens can further compromise the integrity and function of both the intestinal mucus barrier and epithelial barrier. They directly or indirectly reduce interepithelial adhesion, and may even invade and transmigrate across epithelial cells, thereby exacerbating intestinal “leakiness.” For instance, *P.g* employs its gingipains to directly degrade tight junction proteins (e.g., occludin) and adherens junction proteins (e.g., E-cadherin) (Tsuzuno et al., 2021). Certain pathogenic bacteria (e.g., *P.g*, *A.a*, and *T.denticola*) also downregulate E-cadherin expression on intestinal epithelial surfaces, disrupting adherens junctions and facilitating bacterial translocation (Devaux et al., 2019). Moreover, colonization by periodontal bacteria impairs the gut’s immune barrier. For example, P. gingivalis colonization increases pro-inflammatory cytokines (e.g., IL-6 and TNF-α) in the gut (Lee et al., 2022). In summary, periodontal pathogens disrupt the homeostasis of both the gut microbiota and immune barrier, potentially triggering excessive intestinal immune responses, damaging the intestinal barrier, and ultimately contributing to systemic diseases associated with altered gut immunity (Liu et al., 2021).

### 3.2 Gut dysbiosis affects oral homeostasis

**Figure 2:**
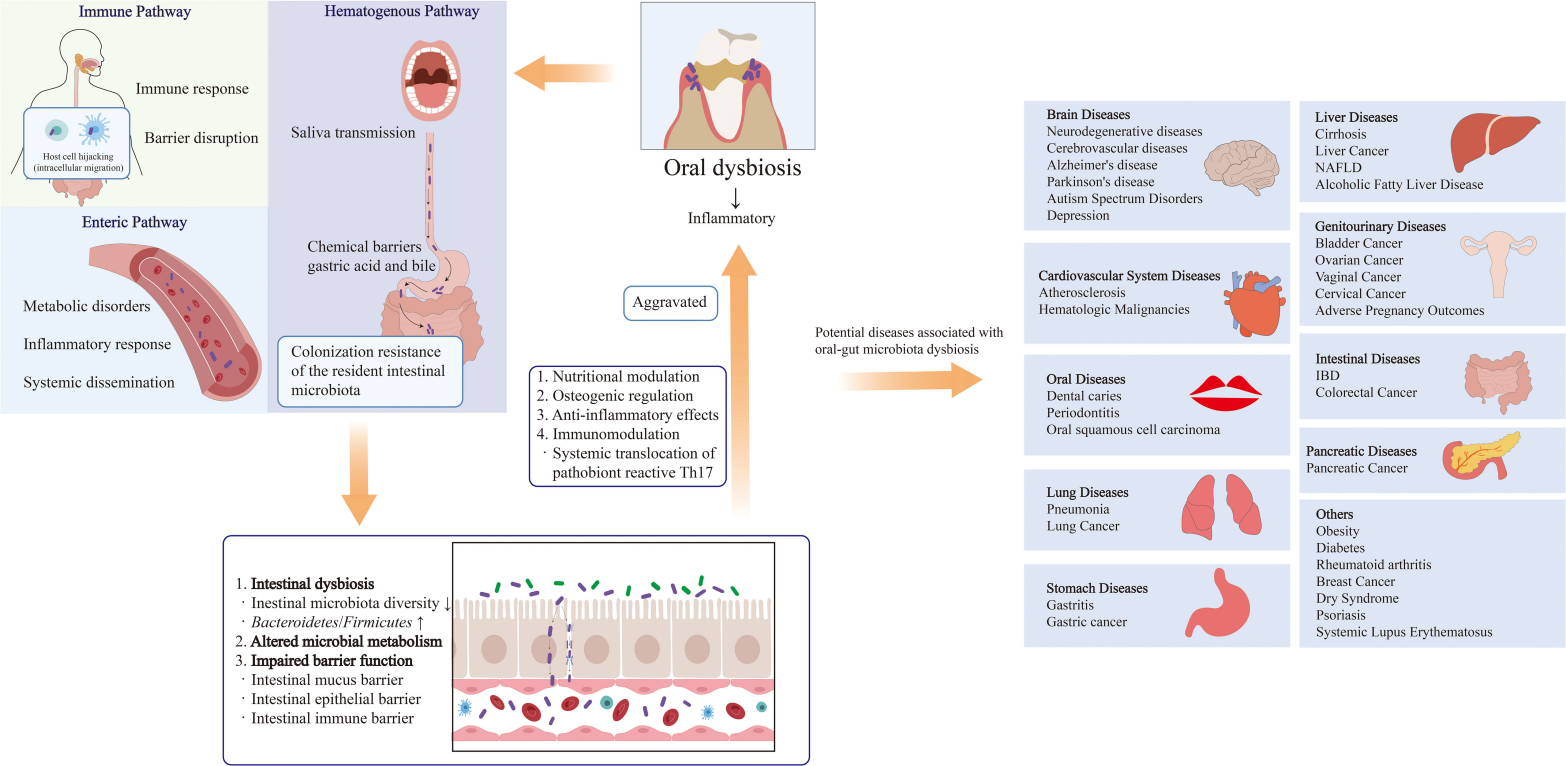
Bidirectional interactions between the oral and gut microbiota and their influencing factors. The oral microbiota can influence the composition and function of the gut microbiota through the following pathways: (1) Enteric route: Oral microbiota are transported to the gut via daily saliva swallowing. (2) Hematogenous route: Oral microbiota can enter systemic circulation and disseminate to the gut via the bloodstream. (3) Immune cell migration route: Certain oral bacteria can survive within immune cells and migrate from the oral mucosa to the gut mucosa alongside these cells. The gut microbiota can modulate the oral microbiota through the following mechanisms: (1) Nutritional metabolism regulation: Influencing host nutrient absorption and distribution. (2) Bone metabolism regulation: Modulating the host’s osteogenesis/osteoclast balance. (3) Anti-inflammatory effects: Producing anti-inflammatory metabolites. (4) Immune regulation: Regulating systemic and local immune responses.

A healthy gut microbiota plays a dual role in maintaining gut homeostasis and ameliorating oral dysbiosis, thereby contributing to periodontal disease control. Conversely, gut dysbiosis and inflammation can adversely affect the composition and stability of oral microbiota. Substantial evidence indicates that patients with IBD exhibit significant oral microbiota alterations that correlate with disease activity and increased susceptibility to oral pathologies (Zhou et al., 2023). Comparative analyses reveal distinct microbiota shifts in IBD patients, including elevated abundances of *Veillonella* and *Prevotella* in saliva (Abdelbary et al., 2022). Furthermore, Elmaghrawy et al. demonstrated IBD-associated perturbations in oral *Firmicutes*, characterized by decreased *Clostridia* and increased *Bacilli*-changes that directly correlate with disease severity (Elmaghrawy et al., 2023). Notably, therapeutic management of IBD can reverse these oral dysbiotic patterns, underscoring the dynamic interplay between gut health and oral microbiota. Emerging research elucidates multiple mechanisms through which gut microbiota and their metabolites influence periodontal pathogenesis: (1) Nutritional modulation: Enhanced Ca²^+^ and Mg²^+^ absorption promotes periodontal tissue integrity. (2) Osteogenic regulation: Direct activation of osteoblasts coupled with inhibition of osteoclast differentiation. (3) Anti-inflammatory effects: Lipocalin and vitamin D supplementation mitigate periodontal inflammation via gut repair. (4) Immunomodulation: Nagao et al. identified a gut-orchestrated immune pathway where oral *P.g* primes Th17 cell activation in Peyer’s patches, with subsequent migration to periodontal tissues (Nagao et al., 2022). The gut-oral axis is further evidenced by Th17/Treg dysregulation. Increased gut permeability and dysbiosis-induced inflammation disrupt bone marrow Th17/Treg homeostasis, exacerbating alveolar bone loss in periodontal and periapical diseases (Jia et al., 2024). Notably, probiotic administration can restore this balance and significantly attenuate bone resorption (Guo et al., 2023). These findings collectively highlight: Bidirectional communication between gut and oral ecosystems, the gut microbiota’s systemic immunomodulatory capacity, therapeutic potential of microbiota-targeted interventions and the critical importance of gut homeostasis for oral health maintenance (**Figure 2**).

## 4 Changes in oral microbiota and systemic diseases

### 4.1 The role of oral-gut axis in the digestive system

#### 4.1.1 CRC

Current research indicates that oral pathogens may translocate to the intestine through hematogenous spread, lymphatic dissemination, and by hijacking dendritic cells and macrophages as transport vectors, ultimately leading to ectopic colonization and contributing to the initiation and progression of CRC. The gut microbiota of CRC patients exhibits significant enrichment of oral-associated bacteria, including *F.n*, *Parvimonas micra*, *Peptostreptococcus stomatis*, *Peptostreptococcus anaerobius*, and *Solobacterium moorei*. These pathogens demonstrate strong associations with CRC pathogenesis and progression (Yachida et al., 2019). Comparative analyses of fecal samples from healthy individuals and advanced CRC patients reveal increased microbiota diversity in CRC, particularly involving 10 oral bacterial species such as *F.n*, *Streptococcus mitis*, and *Haemophilus parainfluenzae (* Loftus et al., 2021). Experimental evidence further supports this connection, as fecal microbiota transplantation from PD patients to CRC mouse models promotes tumorigenesis through oral-gut microbiota remodeling (Shi et al., 2023).

Among these ectopically colonized oral pathogens, *F.n* has been identified as a potential oncogenic driver in CRC (Casasanta et al., 2020). *F.n* employs multiple virulence mechanisms: its adhesin Fap2 facilitates CRC cell invasion and induces pro-inflammatory cytokines IL-8/CXCL1 to promote proliferation and metastasis, while simultaneously activating TIGIT to suppress T/NK cell activity and enable immune evasion (Gur et al., 2015). The alternative adhesin FadA activates the E-cadherin/β-catenin pathway, upregulating CHK2 to induce DNA damage and driving oncogene expression through Wnt/β-catenin signaling (Rubinstein et al., 2013; Guo et al., 2020). Furthermore, *F.n* promotes M2 macrophage polarization via TLR4/NF-κB/S100A9 signaling and confers 5-FU chemoresistance by upregulating BIRC3, establishing itself as an independent risk factor for advanced CRC recurrence (Zhang et al., 2019; Hu et al., 2021). These findings collectively position *F.n* as a multifaceted contributor to CRC progression and a promising therapeutic target.

#### 4.1.2 NAFLD

Kuroe et al. conducted a 5-year follow-up study involving 34 Japanese subjects diagnosed with NAFLD but without liver fibrosis at baseline, examining the potential influence of PD on disease progression. Their findings revealed that individuals with moderate to severe PD faced a significantly higher risk of NAFLD advancing to fibrotic liver injury (odds ratio [OR] 1.82, 95% confidence interval [CI] 0.94–3.49, p = 0.074) (Kuroe et al., 2021). Further supporting this association, Nakahara et al. demonstrated a significant correlation between liver fibrosis progression and the antibody titer of *P.g* fimA genotype 4 (P=0.0081), suggesting that this periodontal pathogen may play a regulatory role in the transition from NAFLD to non-alcoholic steatohepatitis (NASH) (Nakahara et al., 2018).

Growing evidence indicates that the oral microbiota, including *P.g*, can translocate to the gut in patients with liver disease (Qin et al., 2014; Dubinkina et al., 2017; Åberg and Helenius-Hietala, 2022). Upon entering the gastrointestinal tract, these microbiota may disrupt the gut microbiota composition, increase gut permeability, and contribute to endotoxemia-associated liver injury. Additionally, gut dysbiosis can elevate hepatotoxic substances such as endotoxins and ethanol while impairing bile acid metabolism and signaling, further exacerbating hepatic damage (Kuraji et al., 2023). This mechanism is supported by Arimatsu et al., who observed that mice orally administered *P.g* exhibited a marked increase in gut *Bacteroidetes*, elevated blood endotoxin levels, and reduced ileal tight junction protein expression, alongside insulin resistance and altered gene expression in adipose and liver tissues (Arimatsu et al., 2014). Similarly, Sasaki et al. found that intravenous injection of ultrasound-treated *P.g* in high-fat diet-fed mice led to impaired glucose tolerance, insulin resistance, hepatic steatosis, and gut microbiota shifts—specifically, a decline in *Ruminococcaceae* and an increase in *Lactobacillus johnsonii* and *Lactobacillus reuteri (* Sasaki et al., 2018). Notably, these *P.g*-induced gut dysbiosis patterns align with clinical features observed in many chronic liver diseases (CLD).

**Figure 3:**
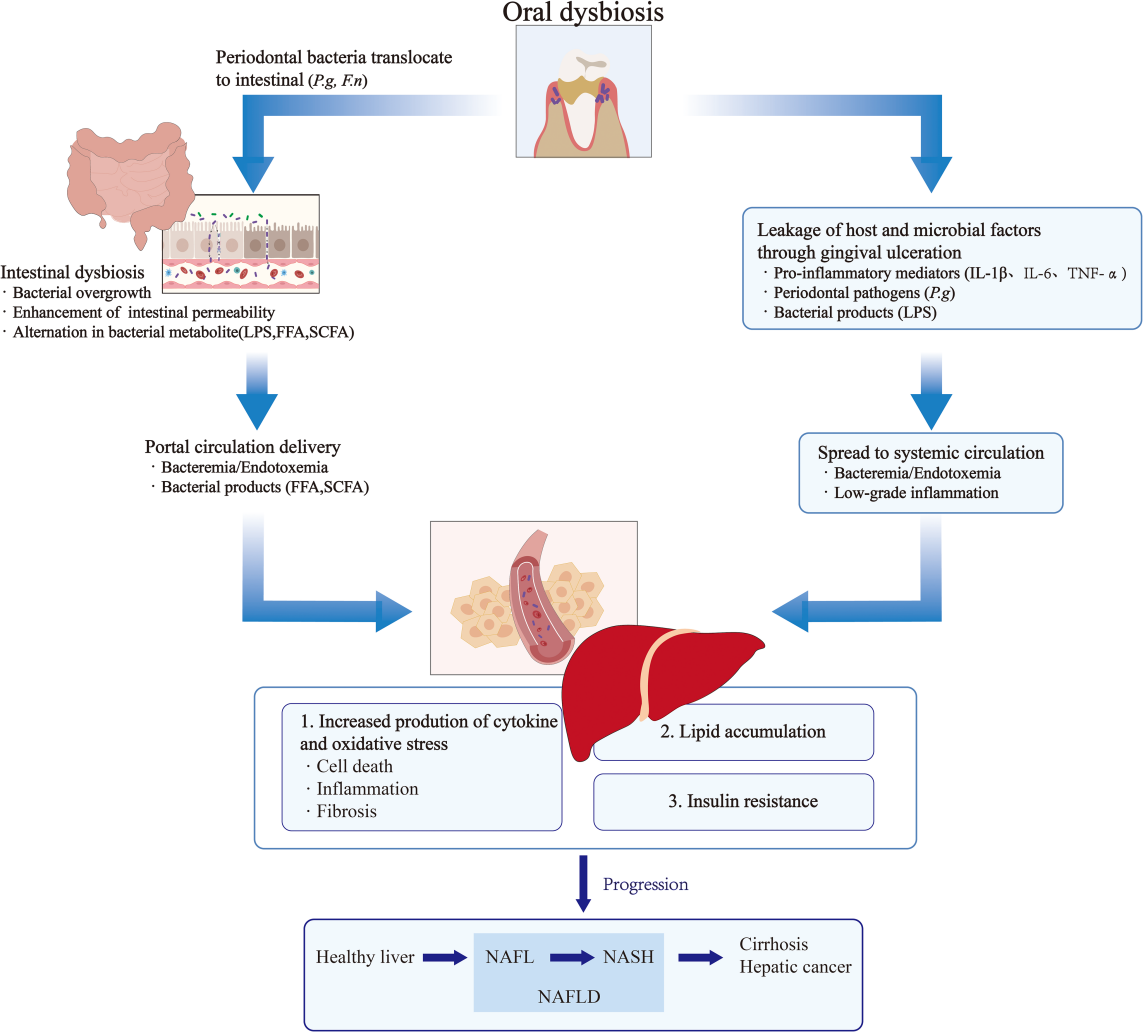
Oral and gut microbiota and their metabolites can reach the liver through hematogenous dissemination or via the gut-liver axis translocation pathway. By mechanisms of insulin resistance, lipid metabolism dysregulation, and inflammatory activation, they promote the progression of liver diseases. These pathophysiological alterations collectively constitute key driving factors in the progression from nonalcoholic fatty liver disease (NAFLD) to nonalcoholic steatohepatitis (NASH) and hepatic fibrosis.

The oral microbiota may directly influence metabolic dysfunction-associated NAFLD through hematogenous dissemination. Taking *P.g* as an example, this pathogen and its virulence factors (including fimbriae, LPS, and gingipains) can reach the liver via systemic circulation, where they bind to specific receptors to induce inflammatory immune responses and activate intracellular signaling pathways (Wang T. et al., 2022). Upon binding to host cells and tissues, *P.g* not only stimulates immune-inflammatory cascades but also evades host immune clearance by modulating the complement system, thereby facilitating bacterial colonization and persistence within host tissues. Furthermore, *P.g* impairs insulin signaling by reducing insulin receptor substrate-1 (IRS-1) and Akt/GSK-3β phosphorylation rates, severely suppressing glycogen synthesis. This metabolic disruption may also be mediated by gingipain activity, which interferes with glucose homeostasis (Ishikawa et al., 2013; Seyama et al., 2020). Fimbriae, particularly the FimA protein, contribute to NAFLD progression by binding to TLR2, CR3, and CXCR4, thereby inducing diverse immune-inflammatory responses. Additionally, *P.g*-derived LPS promotes intracellular lipid accumulation and upregulates pro-inflammatory cytokines, including IL-1, IL-8, and TNF-α, via nuclear factor-κB (NF-κB) and c-Jun N-terminal kinase (JNK) signaling pathways (Ding et al., 2019). Other bacterial components, such as gingipains, biofilms, and outer membrane vesicles, further exacerbate NAFLD by enhancing bacterial survival and facilitating hepatic translocation (Wang T. et al., 2022). Collectively, these findings demonstrate that *P.g* exerts direct pathogenic effects on NAFLD development through multiple virulence mechanisms. In summary, specific periodontal pathogens, particularly *P.g*, may play a crucial role in the pathogenesis and progression of liver diseases by modulating the oral-gut-liver axis (**Figures 2**, **3**).

### 4.2 The role of oral-gut axis in the nervous system

AD is a progressive neurodegenerative disorder and the leading cause of cognitive and behavioral impairment worldwide. Its neuropathological hallmarks include amyloid-beta (Aβ) plaques and neurofibrillary tangles mediated by phosphorylated tau. Noble et al. (2009) analyzed data from the Third National Health and Nutrition Examination Survey (NHANES-III) and reported that individuals with elevated serum *P.g* IgG levels exhibited a higher likelihood of impaired speech memory and performance in subtraction tests (Noble et al., 2009). Furthermore, periodontal pathogens such as *T.denticola*, *Tannerella forsythia*, and *P.g* have been detected in postmortem brain tissue of AD patients (Poole et al., 2013; Dominy et al., 2019). The research findings by Lu et al. demonstrate that periodontitis-associated salivary microbiota may exacerbate the pathogenesis of Alzheimer’s disease (AD) through gut-brain axis crosstalk (Lu et al., 2022). Chen et al. investigated the composition of oral and gut microbiota across different stages of AD and observed a progressive increase in oral *Firmicutes* and *Clostridiales* abundance, while gut *Firmicutes* and *Bacteroidetes* levels declined from healthy controls to mild and moderate AD cases. Notably, the overlap between oral and gut microbiota intensified with AD severity, suggesting that moderate AD patients exhibit a higher propensity for oral-to-gut microbiota transmission compared to mild AD patients or healthy controls (Chen et al., 2022).

**Figure 4:**
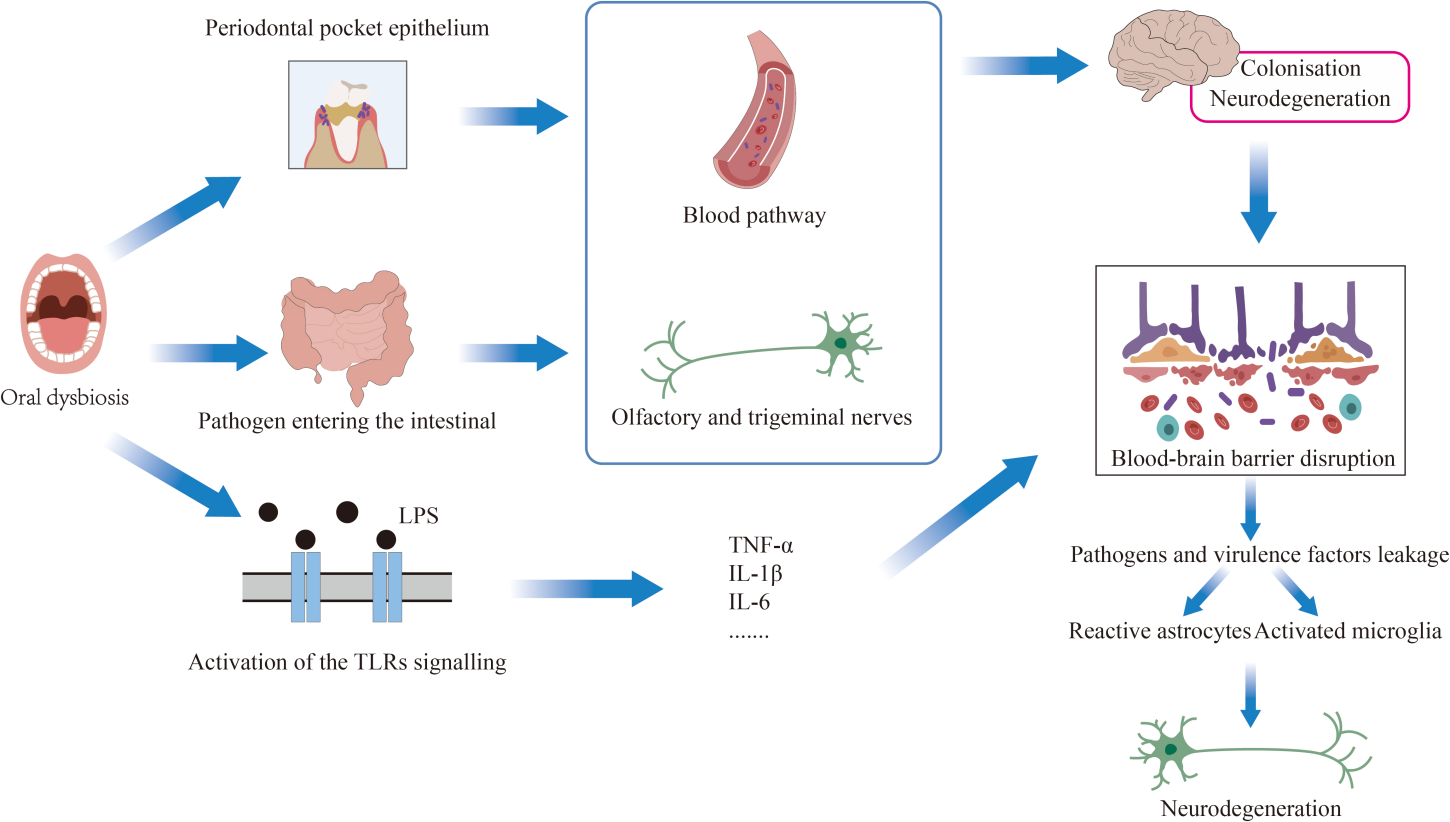
Current research indicates that periodontitis can induce neuroinflammation through multiple pathways, thereby promoting the onset and progression of neurodegenerative diseases. Oral pathogens primarily affect the central nervous system through three mechanisms: (1) systemic inflammatory pathways, (2) oral-gut-brain axis pathways, and (3) neural transmission pathways.

Oral microbiota may reach the brain through several potential pathways. First, via direct oral-brain communication, where periodontal pathogens and their virulence factors can compromise the blood-brain barrier (BBB) integrity by increasing its permeability, thereby gaining access to and damaging central nervous system tissue (Sansores-España et al., 2021). Second, through trigeminal nerve translocation, as demonstrated by Santiago Tirado et al., showing that certain periodontal pathogens like *P.g* employ virulence factors to evade phage lysosome formation, enabling intracellular survival and subsequent axonal/dendritic migration via Trojan horse mechanisms or vesicular transport (Santiago-Tirado et al., 2017). Third, the oral-gut-brain axis provides another route, wherein AD-associated gut dysbiosis increases gut barrier permeability, facilitating microbiota metabolite transfer into circulation. These circulating factors can then activate central nervous system(CNS) immune cells, triggering neuroinflammation and neuronal loss characteristic of AD (Bello-Corral et al., 2023). Enhanced gut leakage through this axis allows systemic dissemination of virulence factors and pro-inflammatory mediators either through circulation or via vagus nerve fibers (Sansores-España et al., 2021). The gut microbiota further modulates BBB function through multiple mechanisms, including secretion of hormones, metabolic cofactors, GI-derived small molecules, and inflammatory mediators like cytokines that influence BBB permeability through oxidative stress pathways. Chronic periodontal disease driven by dysbiosis promotes neuroinflammation and cognitive impairment through partial activation of the TLR4/MyD88/NF-κB signaling cascade, providing mechanistic support for the oral-gut-brain axis (Xue et al., 2020; Li et al., 2022). Compellingly, recent murine studies demonstrate that oral *P.g* inoculation induces both cognitive deficits and gut dysbiosis, offering direct experimental evidence for this pathway (Chi et al., 2021). In summary, oral bacteria can achieve ectopic colonization in the central nervous system through the oral-brain bidirectional communication and the oral-gut-brain axis pathways (**Figure 4**).

Current research reveals that ectopically colonized oral pathogens in the CNS can trigger excessive β-amyloid (Aβ) accumulation. These BBB-penetrating oral microbita activate neuroimmune responses, which not only enhance Aβ production but may also induce Aβ cascade amplification, thereby accelerating AD pathogenesis (Weaver, 2020). Taking *P.g* as an example, this pathogen stimulates aberrant β-amyloid generation through activating the cathepsin B (CatB)/NF-κB signaling pathway while upregulating TLR-2 and IL-1β expression (Nie et al., 2019). Furthermore, *P.g*-derived outer membrane vesicles (OMVs) containing gingipains not only boost Aβ production but also generate LPS via TLR4-mediated NF-κB and MAPK pathway activation, directly contributing to Aβ plaque formation (Zhao et al., 2017; Dominy et al., 2019). Beyond amyloid pathology, periodontal pathogens promote tau hyperphosphorylation, another hallmark of AD progression (Narengaowa et al., 2021). These microbiota also exacerbate neuroinflammation through neuromodulatory mechanisms: *P.g*-derived LPS elevates IL-17A levels in splenic mononuclear cells, and this cytokine can breach the blood-brain barrier to activate microglia-mediated neuroinflammation and cognitive impairment (Sun et al., 2015). Feng et al. demonstrated that serum IL-17A promotes neuronal apoptosis through IL-17RA interaction in *P.g*-exposed R1441G mice while *in vitro* studies confirm IL-17A’s capacity to activate microglia and accelerate dopaminergic neuron degeneration (Chen et al., 2020; Feng et al., 2020). Supporting these findings, Dominy et al. identified *P.g* DNA and gingipain antigens in AD mouse cerebrospinal fluid, confirming that periodontal pathogens induce neuroinflammation and neurodegeneration through neuronal pyroptosis and caspase-1 activation, resulting in elevated neuroinflammatory cytokines IL-1β and IL-18. Notably, small-molecule gingipain inhibitors have shown therapeutic potential by blocking *P.g*-induced neurodegeneration, providing neuroprotection and significantly reducing cerebral *P.g* burden in murine models (Dominy et al., 2019).

### 4.3 The role of oral-gut axis in the reproductive system

**Figure 5:**
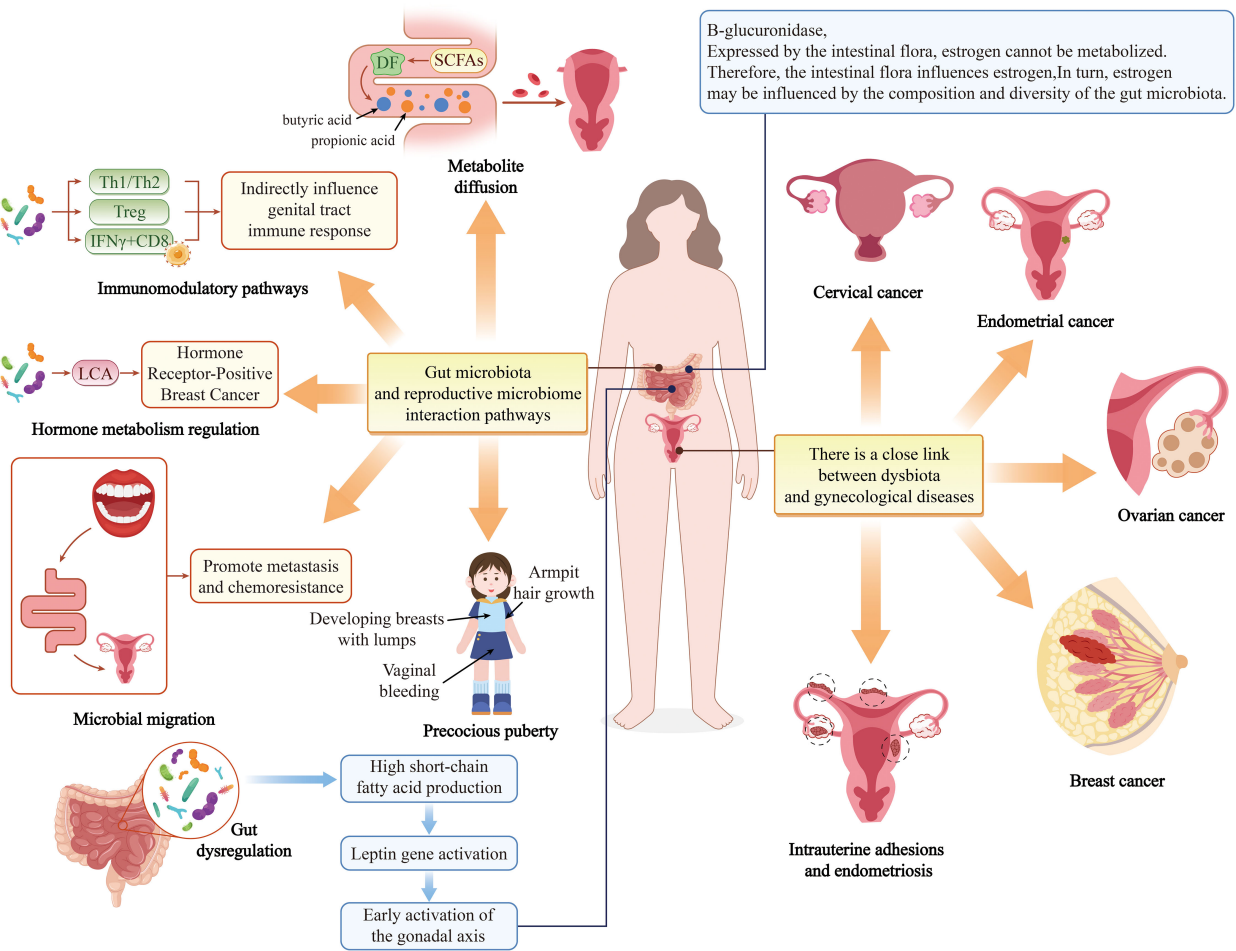
The impact of oral-gut microbiota on the female reproductive system.

Emerging evidence highlights a strong association between disruptions in the oral and gut microbiota and various health conditions, including breast cancer (BC), adverse pregnancy outcomes (APOs), and gynecological disorders (GDs) such as preterm labor, spontaneous abortion, endometriosis, bacterial vaginosis (BV), and ovarian and cervical cancers (CC) (Ghosh et al., 2024) (**Figure 5**).

Petricevic et al. demonstrated that 80% of pregnant women and 40% of postmenopausal women shared identical *lactic acid bacteria* (*LAB*) isolates between their vaginal and rectal microbiota. Furthermore, 53% of pregnant women and 33% of postmenopausal women exhibited overlapping *LAB* strains in oral and rectal samples, suggesting that the oral and gut tracts may serve as reservoirs for vaginal colonization (Petricevic et al., 2012). Adolescent women with suboptimal vaginal microbiota exhibited a higher prevalence of periodontal pathogens—including *Prevotella intermedia* and *P.g*—in their supragingival microbiota, alongside enriched *Pseudomonas aeruginosa* and *Pseudomonas intermedia* in saliva (Balle et al., 2020). Notably, *Prevotella copri*, a gut microbe associated with a healthy microbiota, was found exclusively in vaginal samples of women without BV. Wei et al. investigated the vaginal and oral microbiota in women with a history of abortion, HPV infection, or CC, revealing a significant increase in periodontal pathogens among CC patients. Shared pathogens such as *Porphyromonas* spp. and *Prevotella* spp. were identified as oral biomarkers for CC. Their findings underscore that vaginal and oral microbiota are interconnected, with microbiota translocation between body sites potentially influencing systemic metabolism (Zhang et al., 2024). Huang et al. quantified *F.n* levels in tumor tissues from 112 CC patients using qPCR, revealing significantly elevated *F.n* in CC tissues, particularly in recurrent cases. A multi-omics study by Maarsingh et al. further demonstrated that *F.n* infection exacerbates HPV persistence and cervical neoplasia by inducing pro-inflammatory responses, upregulating cell cycle metabolism, and modulating oxidative stress and lipid metabolism—key drivers of tumorigenesis. Additionally, CC cells with high intratumoral *F.n* exhibited cancer stem cell (CSC)-like properties, implicating oral and gut microbiota in CC progression (Maarsingh et al., 2022).

Large-scale epidemiological studies support a link between PD and BC. A U.S. cohort study of 65,869 postmenopausal women found that PD significantly increased BC risk (adjusted hazard ratio [aHR] = 1.13, 95% CI: 1.03–1.23) (Nwizu et al., 2017). A meta-analysis of 16,811 participants further confirmed this association (RR = 1.18, 95% CI: 1.11–1.26, I² = 17.6%) (Shi et al., 2018). Parhi et al. discovered that oral *F.n* can translocate hematogenously to colonize breast tissue milk ducts. BC tissues express high levels of D-galactose-beta [1-3]-N-acetyl-D-galactosamine (Gal-GalNAc, or T-antigen), which facilitates *F.n* adhesion via its surface lectin Fap2. *F.n* may drive tumor growth and metastasis by suppressing T-cell infiltration and upregulating matrix metalloproteinase-9 (MMP-9). Notably, metronidazole treatment attenuated breast tumor metastasis in murine models, highlighting Fap2 as a potential therapeutic target (Narengaowa et al., 2021). Clinical studies report elevated *F.n* abundance in breast tumors, with bacterial load correlating positively with tumor size and disease progression (Parhi et al., 2020; Parida and Sharma, 2020; Li et al., 2023; Baima et al., 2024b). Recent *in vivo* work confirmed that *F.n*-derived small extracellular vesicles (*F.n*-EVs) promote BC cell proliferation, migration, and invasion, accelerating tumor growth and metastasis. The gut microbiota also influences BC pathogenesis. Multiple studies implicate gut microbiota and their metabolites in BC initiation and progression through mechanisms including: epithelial-mesenchymal transition (EMT), invasive phenotype acquisition, DNA damage and epigenetic modifications, microenvironmental inflammation, estrogen and short-chain fatty acid (SCFA) production (Haque et al., 2022; Wang N. et al., 2022; Álvarez-Mercado et al., 2023; Yang et al., 2023). Zheng et al. observed marked dysbiosis in the oral and gut microbiota of dogs with canine mammary tumors (CMTs), characterized by elevated *Bacteroidetes*—a microbial signature shared among oral, gut, and intratumoral microbiota. These findings suggest a potential pathway for microbiota dissemination from the oral cavity to the gut and, ultimately, to distant breast tumors (Zheng et al., 2022).

## 5 Discuss

The accessibility of oral sampling presents a unique advantage for developing non-invasive screening protocols. This approach could revolutionize early detection paradigms for systemic diseases while enabling longitudinal monitoring of disease progression. Emerging research has established the oral microbiota as a promising non-invasive biomarker for early detection of various systemic diseases, including gastrointestinal and hepatic malignancies (Torres et al., 2015). The study by Huang et al. pointed out, the predictive value of salivary microbiota composition for gastric cancer (GC) and its premalignant stages. Their findings revealed significant depletion of multiple bacterial taxa in GC patients compared to those with chronic gastritis (SG) or acute gastritis (AG), including *Bulleidia Fusobacterium*,*Haemophilus*,*Lachnoanaerobaculum*,*Neisseria*,*Parvimonas*,*Peptostreptococcus*,*Porphyromonas* and *Prevotella (* Huang et al., 2021). Further supporting this concept, Peters et al. employed 16S rRNA gene sequencing to identify specific oral microbiota signatures associated with esophageal cancers. Their analysis demonstrated that Tannerella forsythia correlated with increased risk of esophageal adenocarcinoma (EAC), while *P.g* showed association with esophageal squamous cell carcinoma (ESCC), suggesting distinct microbiota etiologies for these malignancies (Peters et al., 2017).In hepatobiliary malignancies, Lu et al.’s investigation of tongue-coating microbiota through 16S rRNA sequencing revealed significant dysbiosis in hepatocellular carcinoma (HCC) patients. Notably, *Oribacterium* and *Fusobacterium* emerged as particularly discriminative taxa, exhibiting potential as specific biomarkers for HCC detection (Lu et al., 2016). Although the Zürcher research team has demonstrated that human saliva can serve as a diagnostic sample for multiple diseases, its clinical application is limited by the high variability of salivary composition. This variability primarily stems from interference by various exogenous factors, posing challenges to the reliability of saliva-based diagnostics for brain disorders such as Alzheimer’s disease (Zürcher and Humpel, 2023). Consequently, saliva-based diagnostic approaches require stringent control of testing conditions and careful exclusion of confounding comorbidities.

Current research indicates that gut dysbiosis is often accompanied by structural alterations in the oral microbiota, with ectopic colonization of oral-origin bacteria detected at dysbiotic sites. This oral-gut microbiota interaction exhibits a distinct bidirectional characteristic (Qin et al., 2014; Imai et al., 2021). Pietropaoli et al. demonstrated using SAMP1/YitFc mice (a spontaneous model of Crohn’s disease) that this model naturally develops periodontitis, and the severity of periodontitis shows a significant positive correlation with the degree of ileal inflammation (Pietropaoli et al., 2014). Furthermore, findings from Xiao and D’Aiuto et al. confirmed that systemic inflammatory diseases may also compromise the immune barrier function of the oral mucosa, leading to increased inflammatory burden and heightened susceptibility to periodontal disease (Xiao et al., 2017; D’Aiuto et al., 2018; Kunath et al., 2024). In summary, although the precise mechanisms underlying the role of the oral microbiota in systemic diseases remain to be elucidated, existing evidence suggests that this is fundamentally a complex regulatory network involving multiple organs, characterized by continuous microbial migration and dynamic interactions. Importantly, dysbiosis in either the oral or gut microbiota can trigger chronic systemic inflammation, a well-recognized pathogenic driver of many systemic diseases (Kunath et al., 2024). Chen et al.’s findings not only revealed that PD and the oral microbiota—through oral-gut microbial transmission and ectopic colonization of saliva-derived pathogens in the gut—are closely associated with hypertension (HTN), but also proposed an innovative strategy for the combined management of PD and HTN. Specifically, detecting oral-origin microbes in the gut may provide novel targets for early warning and precision intervention in HTN. Supporting evidence comes from Baima et al.’s team, who used 16S rRNA gene amplicon sequencing to analyze fecal and saliva samples from PD patients. Their results confirmed that periodontal treatment not only reduced oral dysbiosis but also altered gut microbiota composition. Kuraji et al.’s research on the antimicrobial peptide Nisin demonstrated its dual ability to mitigate periodontal and intestinal inflammation while promoting a healthier gut-liver microbiota. These findings align with the perspective of Genco et al.’s team, which advocates for the inclusion of routine periodontal assessment and treatment in the comprehensive management of type 2 diabetes.

Currently, gut microbiota therapies (e.g., FMT) have shown great potential in treating extraintestinal diseases, but they neglect the regulation of oral microbiota (**Table 1**). Periodontal treatment alone has demonstrated efficacy, yet its regulatory effects on gut microbiota have not received adequate attention (**Table 2**). Mounting evidence indicates that combined periodontal-intestinal interventions may produce synergistic therapeutic effects against systemic diseases. Moreover, this article elaborates on the profound impact of maintaining periodontal health in reducing the risk of many intestinal and extraintestinal diseases. Based on existing research, we propose that: dynamically monitoring oral-gut microbiota changes, targeting oral microbiota and/or intervening in oral-gut microbial transmission may become novel strategies for preventing and treating both oral and systemic diseases. This study particularly reveals the potential mechanisms by which *P.g* and *F.n* induce systemic diseases through the oral-gut axis. However, the complexity of oral and gut microbiomes suggests that other microbiota (e.g., *Prevotella*, *Streptococcus mutans*, or gut commensal bacteria) may participate in the development of systemic diseases through similar or synergistic pathways. Future research should focus on: (1) applying multi-omics technologies to elucidate the dynamic changes of microbial metabolites in the oral-gut axis and their immune regulation mechanisms; (2) developing experimental models that can trace microbial transmission directions to clarify the patterns of bidirectional oral-gut transmission; (3) blocking pathogenic oral-gut transmission through precision antibiotic therapy, FMT, prebiotic/probiotic applications, and optimized oral hygiene management, thereby providing innovative prevention and treatment solutions for various systemic diseases including metabolic disorders, cardiovascular diseases, and neurological diseases.

**Table 1:** Gut microbiota interventions (e.g., FMT, probiotics) in parenteral diseases.

Nation	Types of research	Sample size	Treatment	Results	Conclusions	References
America	Randomized controlled animal experiments	24 mice	Nisin (an antimicrobial peptide produced by *Lactococcus lactis*)	Nisin treatment mitigated the changes in the brain microbiota composition, diversity, and community structure, and reduced the levels of periodontal pathogen DNA in the brain induced by periodontal disease. In addition, the concentrations of amyloid-β 42 (Aβ42), total Tau, and Tau (pS199) (445.69 ± 120.03, 1420.85 ± 331.40, 137.20 ± 36.01) were significantly higher in the infection group compared to the control group (193.01 ± 31.82, 384.27 ± 363.93, 6.09 ± 10.85), respectively. Nisin treatment markedly reduced the Aβ42 (261.80 ± 52.50), total Tau (865.37 ± 304.93), and phosphorylated Tau (82.53 ± 15.77) deposition in the brain of the infection group.	Nisin abrogation of brain microbiota dysbiosis induces beneficial effects on AD-like pathogenic changes and neuroinflammation, and thereby may serve as a potential therapeutic for periodontal-dysbiosis-related AD.	(Zhao et al., 2023)
China	Randomized, placebo-controlled trials	56 patients	FMT	During the follow-up, no severe adverse effect was observed, and patients with FMT treatment showed significant improvement in PD-related autonomic symptoms compared with the placebo group at the end of this trial (MDS-UPDRS total score, group×time effect, B = -6.56 [−12.98, −0.13], *P* < 0.05)	Oral FMT capsules can be safely and feasibly engrafted in these patients, demonstrating the promising potential of FMT in improving current PD medications. And the study also suggested that gut microbiota could serve as a biomarker to predict the response to FMT intervention in PD.	(Cheng et al., 2023)
China	Randomized and controlled clinical trial	75 patients	Non-FMT group(a *Bifidobacterium* viable preparation and *Lactobacillus acidophilus* capsules),and FMT group	The hepatic fat attenuation evaluated by FibroScan was significantly reduced after FMT (*p* < 0.05). Compared to healthy individuals, patients with NAFLD before the FMT had lower Chaol indexes [prior to FMT (pri-FMT) vs. healthy group, *p* < 0.05], suggesting a lower abundance of the gut microbiota in patients with NAFLD. On the other hand, there was no statistical difference in the Chaol indexes between NALFD patients post-FMT (po-FMT) and healthy individuals (*p* > 0.05), indicating that the impaired abundance of the gut microbiota had been improved after FMT.	FMT can improve NAFLD by balancing gut microbiota disorder. FMT had better effects on the improvement of lean than of obese NAFLD patients.	(Xue et al., 2022)
China	A single-center randomized controlled clinical trialand animal testing	20 patients and several mice	Lactobacillus acidophilus(*L.acidophilus)*	A significant decrease in blood liver function indexes after *L.acidophilus* treatment, indicating prompt liver injury recovery progression. Similar to the results in mice, the BAs profiles after *L.acidophilus* treatment in patients showed relatively higher unconjugated BAs and enhanced BAs excretion.	In cholestasis patients, supplementation of *L. acidophilus* promoted the recovery of liver function and negatively correlated with liver function indicators, possibly in relationship with the changes in BAs profiles and gut microbiota composition.	(Wu et al., 2024)
India	A Randomized, Double-Blind, Placebo-Controlled Trial	174 patients	Placebo (G1), oral probiotic (G2), vaginal probiotic (G3), and probiotic combination (oral lactic acid bacteria and bifidobacteria + vaginal lactobacilli)	The incidence of UTI at 4 months in G1, G2, G3, and G4 was 70.4%, 61.3%, 40.9%, and 31.8%, respectively. The mean number of symptomatic UTI recurrences at 4 months was significantly lower (P <.05) in G3 (1.06) and G4 (1.07) compared with G1 (2.1) and G2 (1.63). Further, the time to first symptomatic UTI (days) was significantly longer (P <.05) in G3 (123.8) and G4 (141.8) compared with G1 (69.3) and G2 (71.9). Probiotic supplementations were well tolerated with no serious adverse events.	Prophylactic supplementation with either vaginal probiotics or in combination with oral probiotics demonstrated effectiveness in preventing recurrent symptomatic UTI episodes.	(Gupta et al., 2024)

**Table 2:** Periodontal therapy in systemic diseases.

Nation	Types of research	Sample size	Treatment	Results	Conclusions	References
Australian	Randomized controlled trial	273 patients	Periodontal debridement	Intima-media thickness decreased significantly after 12 months in the intervention group (mean reduction=−0.023 [95% confidence interval, −0.038 to −0.008] mm) but not in the control group (mean increase=0.002 [95% CI, −0.017 to 0.022] mm). The difference in intima-media thickness change between treatment groups was statistically significant (−0.026 [95% CI, −0.048 to −0.003] mm; P=0.03). In contrast, there were no significant differences between treatment groups in pulse wave velocity at 3 months (mean difference, 0.06 [95% CI, −0.17 to 0.29] m/s; P=0.594) or 12 months (mean difference, 0.21 [95% CI, −0.01 to 0.43] m/s; P=0.062).	Periodontal therapy reduced subclinical arterial thickness but not function in Aboriginal Australians with periodontal disease, suggesting periodontal disease and atherosclerosis are significantly associated.	(Kapellas et al., 2014)
The UK	Single-center, parallel-group, investigator-masked, randomized trial	264 patients	Intensive periodontal treatment (IPT; whole mouth subgingival scaling, surgical periodontal therapy), and supportive periodontal therapy or control periodontal treatment (CPT; supra-gingival scaling and polishing)	At baseline, mean HbA1cwas 8.1% (SD 1.7) in both groups. After 12 months, unadjusted mean HbA1cwas 8.3% (SE 0.2) in the CPT group and 7.8% (0.2) in the IPT group; with adjustment for baseline HbA1c, age, sex, ethnicity, smoking status, duration of diabetes, and BMI, HbA1cwas 0.6% (95% CI 0.3-0.9; p<0.0001) lower in the IPT group than in the CPT group.	Routine oral health assessment and treatment of PD could be important for effective management of type 2 diabetes.	(D’Aiuto et al., 2018)
Italy	Randomized controlled trial	94 patients	Periodontal therapy	β-Diversity of gut microbiota profiles of PD patients before treatment significantly differed from healthy controls (P < 0.001). Periodontal therapy was associated with a significant change in gut microbiota (P < 0.001), with posttreatment microbiota profiles similar to healthy volunteers. A higher abundance of Bacteroides, Faecalibacterium, Fusobacterium, and Lachnospiraceae was noted in fecal samples of PD patients at baseline compared to healthy controls.	Periodontal therapy both reduces oral dysbiosis and alters gut microbiota composition.	(Baima et al., 2024a)
Japan	Multicenter randomized controlled study	40 patients	Scaling and root planning [SRP; n = 20] and tooth brushing [n = 20] groups	A significantly higher decrease in absolute alanine aminotransferase levels and *P.g* IgG antibody titers in the SRP group than in the tooth brushing group (-12 vs 1 U/L; mean difference [δ], -12; 95% confidence interval [CI], -20 to -5; P = 0.002). The decrease in *P.g* IgG antibody titer was significantly higher in the SRP group than in the tooth brushing group (FDC381, -1.6 [2.5]; δ, -1.6; 95% CI, -2.7 to -0.4; P = 0.0092; SU63, -1.7 [2.0]; δ, -1.7; 95% CI, -2.7 to -0.7). No life-threatening events or treatment-related deaths occurred.	Periodontal treatment induced significant short-term and mid-term reductions in liver enzyme levels and antibody titers. Further research is warranted to clearly define SRP efficacy and tolerability in patients with NAFLD and PD.	(Kamata et al., 2022)
America	A nonrandomized, open-label trial	50 patients	Periodontal therapy, consisted of prophylaxis or scaling and root planing followed by oral hygiene instructions	Cirrhotics, especially those with hepatic encephalopathy (HE), demonstrated improved dysbiosis in stool and saliva, and improved endotoxin, LBP, and salivary and serum inflammatory mediators following periodontal therapy.	Cirrhotic patients who underwent systemic periodontal therapy improved endotoxemia as well as systemic and local inflammation and modulated salivary and fecal microbiota dysbiosis within 30 days. In contrast, endotoxin and lipopolysaccharide-binding proteins were increased over the same duration in the cirrhotic group that did not receive periodontal therapy.	(Bajaj et al., 2018)
